# The outlandish, the realistic, and the real: contextual manipulation and agent role effects in trolley problems

**DOI:** 10.3389/fpsyg.2014.00035

**Published:** 2014-01-30

**Authors:** Natalie Gold, Briony D. Pulford, Andrew M. Colman

**Affiliations:** ^1^Philosophy Department, King's College LondonLondon, UK; ^2^School of Psychology, University of LeicesterLeicester, UK

**Keywords:** context effects, decision making, hypothetical scenarios, responsibility, trolley problems

## Abstract

Hypothetical trolley problems are widely used to elicit moral intuitions, which are employed in the development of moral theory and the psychological study of moral judgments. The scenarios used are outlandish, and some philosophers and psychologists have questioned whether the judgments made in such unrealistic and unfamiliar scenarios are a reliable basis for theory-building. We present two experiments that investigate whether differences in moral judgment due to the role of the agent, previously found in a standard trolley scenario, persist when the structure of the problem is transplanted to a more familiar context. Our first experiment compares judgments in hypothetical scenarios; our second experiment operationalizes some of those scenarios in the laboratory, allowing us to observe judgments about decisions that are really being made. In the hypothetical experiment, we found that the role effect reversed in our more familiar context, both in judgments about what the actor ought to do and in judgments about the moral rightness of the action. However, in our laboratory experiment, the effects reversed back or disappeared. Among judgments of what the actor ought to do, we found the same role effect as in the standard hypothetical trolley scenario, but the effect of role on moral judgments disappeared.

## Introduction

Psychologists and philosophers use hypothetical dilemmas to elicit moral judgments (e.g., Kamm, 1996; Greene et al., 2001; Rozyman and Baron, 2002; Cushman et al., 2006; Schaich Borg et al., 2006; Waldmann and Dieterich, 2007; Nadelhoffer and Feltz, 2008). Psychologists aim to discover the factors that influence judgments, while philosophers use their intuitions to inform moral theorizing. The scenarios are typically fairly outlandish, involving events that are unlikely to occur in everyday life, and mostly concern life and death decisions. For instance, *trolley problems* are a family of moral dilemmas devised by philosophers in order in order to investigate why it is permissible to cause a harm to one in order to save many in some circumstances but not in others (Foot, 1967; Thomson, 1976, 1985). The paradigm trolley problem is *Side-track*: there is a runaway train that threatens to kill five men on the track ahead. An agent can save the five by switching a lever that will divert the trolley onto a side-track. However, on the side-track is one man, who would be killed. The question is whether it is morally permissible for the agent to save the five and kill the one. Other trolley problems, which are often contrasted to Side-track, vary the details about how the five are saved and the one killed.

In the original version of the trolley problem suggested by Foot (1967), the agent was the driver of the trolley. Thomson changed the agent to a passenger (Thomson, 1976) and later to a bystander (Thomson, 1985). One of the reasons that she gave for the change in role is that, as the “captain of the trolley,” the driver is in a special position, being “charged by the trolley company with responsibility for the safety of his passengers and anyone else who might be harmed by the trolley he drives” (Thomson, 1985, p. 1397). In contrast, the bystander at the switch “is a private person who just happens to be there” (Thomson, 1985; p. 1397). The other reason Thomson gave is that the driver, by driving a trolley into the five, would be killing them. Hence the driver faces a choice between killing five and killing one. However, the other scenarios to which the driver is being compared involve the choice between killing one and letting five die—the predicament that is faced by the passenger and the bystander.

Thomson's bystander is now the paradigm trolley problem, but versions in which a passenger can turn the train onto a side-track have also attracted some attention from philosophers (Quinn, 1989) and psychologists (Hauser et al., 2007). Being a passenger or a bystander might also affect what the agent in the scenario ought to do. Passengers are more involved in the situation than bystanders, for whom doing nothing is, arguably, just staying out of it. Specifically, we might think of *bystanders* as onlookers, who are unexpectedly given the chance to intervene and re-direct a threat, whereas *passengers* are already participants in the situation, without being one of the people who are directly affected by the threat.

Previous experiments show that people's moral judgments about turning the train in Side-track are affected by the agent's role, as a passenger or a bystander. Pulford et al. (2012) found that 84% of subjects judged that it was morally permissible for the agent to turn the train down a side-track when she was a passenger, compared to 65% (significantly fewer) when she was a bystander. The passenger scenario replicated a dilemma from Hauser et al. (2007), which elicited a higher level of agreement that it is morally permissible to turn the train (85%) than their other scenarios, some of which were bystander scenarios—although they did not include a bystander version of Side-track.

Side-track is one of the less outlandish versions of the trolley problem. It has even been known to occur in real life (CNN U.S., 2003). However, it is hardly a familiar occurrence. Another popular version, introduced by Thomson (1985), is *Footbridge*, where the agent can save the five by pushing a large man off a footbridge in front of the train, stopping the train but killing the one. As well as imagining an unusual scenario, responding to the Footbridge dilemma involves suspending disbelief that a large person—even one sometimes described as wearing a backpack—would be solid and massive enough to stop a train. Arguably the most far-fetched trolley problem is Frances Kamm's (1996, p. 154) *Lazy Susan* case, where the five and the one are seated on opposite sides of a giant lazy Susan, which the agent can rotate in order to save the five from the train but, in doing so, puts the one in its path.

Philosophers claim to elicit “common sense intuitions” from these scenarios, which they can use in constructing moral theories (Kamm, 1989; p. 227). Those moral theories are presumably supposed to be applicable to everyday moral decisions. However, Woodward and Allman (2007) argue that reliable judgments are the result of learning processes (which may be implicit) with corrective feedback, where feedback could include the experience of others, historical situations, or learning from cases that are analogous to the situation being assessed. Highly unrealistic cases such as trolley problems do not meet this criterion, and Woodward and Allman caution against their use in moral theorizing.

There are several reasons why there may be differences in performance between unrealistic scenarios and real life. One possibility is that mental processes which are adapted to everyday environments perform poorly when tested in an unusual context. This argument is similar to Gigerenzer's external validity critique of the heuristics and biases literature (Gigerenzer et al., 1999). A second possibility is that unusual scenarios may not elicit normal strategies and thought processes. In real life, moral cognition usually operates swiftly and implicitly, and the “extreme and unfamiliar situations such as those posed by classic moral dilemmas could evoke unusual strategies and thought processes rather than those typically used for common moral judgments” (Knutson et al., 2010; p. 379). This has led some psychologists to argue that ecological validity is crucial for studying moral judgment (Moll et al., 2005).

Most dilemmas used in research on moral judgments involve the causing or preventing of deaths, which is far from most people's everyday experience. Gold et al. (2013) found that the standard pattern of intuitions was preserved in hypothetical scenarios that were analogous to Side-track and Footbridge, but where the outcomes were economic harms, such as loss of a job, income, or property damage. This suggests the possibility of investigating judgments in trolley problems that are more familiar from everyday life. It also raises the possibility of operationalizing trolley problems in the laboratory, with subjects making moral judgments about decisions that are actually being taken, whose outcomes affect the distribution of small economic harms. It is standard to use small economic incentives in behavioral economics, including in the study of games that elicit moral behaviors such as altruism, fairness, trust, cooperation, and reciprocity (e.g., Berg et al., 1995; Fehr and Schmidt, 1999; Bolton and Ockenfels, 2000; Andreoni and Miller, 2002; Andreoni et al., 2002; Fehr and Schmidt, 2006).

We present two experiments designed to test whether intuitions about agent role effects in trolley problems are preserved in more familiar scenarios and in real decision-making situations^1^. We took the decision structure of Side-track, where an agent has the possibility of diverting a threat to five people with the side-effect of harming one, and transplanted it to a scenario involving a game show, a context that is familiar to most people who have watched television. The harm that would befall the one and the five involved loss of money rather than loss of life. In Study 1, we used hypothetical scenarios and we compared role effects in judgments in our game show scenario to those in the standard scenario, where the decision is whether or not to turn a train. In Study 2 we operationalized the game show scenario in the laboratory, allowing us to elicit judgments in real time about a decision that was actually being taken.

## Study 1

We conducted a between-subjects experiment, varying the agent's role in the scenario, *onlooker* vs. *participant*, and the context of the decision. In one condition, we used the standard context of the runaway train. In the others, we changed the context to that of a game show, in which the agent can save five contestants from being knocked out and losing their winnings but, as a side effect, this leads to one other contestant being knocked out. Game shows where contestants are knocked out during the course of the game, and where contestants may have to leave the show forfeiting their winnings, are a familiar staple of television.

As well as comparing the train to a game show, we manipulated the level of the loss in the game show scenarios, comparing the *large game show* scenario, where the contestants stood to lose £200,000 (more than the average price of a house in the UK), to the *small game show* scenario, where the contestants stood to lose £10. We elicited judgments about the rightness of the action and about what the agent should do, and we asked subjects about the agent's responsibility for taking the action as well as about various other factors which may be relevant to moral judgment, and about how believable they found the scenario.

### Methods

#### Subjects

There were 1215 subjects: 359 men, 761 women, and 95 people who did not disclose their gender. Subjects were mainly voluntary visitors to an on-line survey, which they completed in their own time, after following a link to a SurveyGizmo online data collection website. The survey was promoted online, including at http://psych.hanover.edu/research/exponnet.html, and through UK university e-mail lists. There were 31 subjects who voluntarily participated in a pen and paper version distributed in an undergraduate philosophy class at the University of Edinburgh. Subjects were not paid for their participation. The majority of the subjects (67%) were British or American, the rest came from all over the world; 75% spoke English as their native language. Subjects were aged between 18 and 72 years (*M* = 24.87, *SD* = 8.83).

#### Materials

We compared six scenarios in a 2 (Role: *onlooker* vs. *participant*) × 3 (Context: *train* vs. *large game* vs. *small game*) experimental design. The *train* scenarios were based on the standard trolley problem where the agent has the possibility of turning a train onto a side-track, saving five lives at the cost of one. We varied whether the agent was a bystander on the tracks (onlooker) or a passenger on the train (participant in the scenario). Phrases in italics indicate variations between conditions, *onlooker/participant*:

Peter is *taking his daily walk near the train tracks when he sees a runaway train approaching with no driver/a passenger on a train whose driver has just shouted that the brakes have failed, and who then fainted of shock.* The train is moving so fast that anyone it hits will die immediately. There are five people working on the main track. It is obvious that they will not be able to get off the track in time and, if nothing is done, they will be killed.

The track has a side-track leading off to the left. *Peter is standing next to a lever. If he pulls the lever, that will/Peter can* turn the train onto the side track and the five people on the main track will not die. But a person is working on the side track. If the train goes onto the side track, then the person on the side track will die. Peter is aware of all these facts.

Thus, Peter can pull the lever, in which case the one person will die but the five people will not; or Peter can refrain from pulling the lever, in which case the five people will die but the one person will not.

In the *game show* scenarios, we moved the action to a game show and varied whether the agent was an audience member (onlooker) or a contestant (a participant). Phrases in italics indicate variations between conditions (*onlooker/participant*) and *large/small* loss:

Peter is a *member of the studio audience watching/contestant on* a game show. Five contestants have each earned *£200,000/ £10* prize money by answering questions over several rounds, and their tokens are nearing the winning side of the game board. A ball is suddenly released and is rolling toward the tokens of the five contestants and, if nothing is done, they will be knocked out of the game and lose their prize money.

*Peter sees that a button on his armrest has just lit up to indicate that he has been randomly selected by computer to take part in the show.* Peter has the option to press *the/a* button and knock the ball onto another path. But another contestant, who has also earned *£200,000/ £10* prize money, has a token on the new path and will be knocked out of the game and lose his prize money. *Whether or not he presses the button will not affect Peter's winnings.* Peter is aware of all these facts.

#### Procedure

Subjects were randomly allocated to read only one of the six scenarios. After reading the scenario subjects were asked:

1. Is it morally wrong for Peter to *turn the train/press the button?* (Yes/No) and to rate the moral right or wrongness of the action on a seven point scale (-3 *Definitely wrong* to +3 *Definitely right*).
2. Should Peter *pull the lever/press the button?* (Yes/No).
3. To what extent is it Peter's responsibility to *turn the train/press the button*?, rated on a seven point scale (-3 *Not at all* to +3 *Totally*).
4. Assuming that Peter *pulled the lever*/*pressed the button*, to what extent do you agree with the following statements:
  - Peter intended that the *person on the side track would die/contestant with the token on the new path would lose their prize money*
  - Peter is to blame for the death of the *person on the side-track/loss of the prize money of the contestant with the token on the new path*
  - Peter caused the *death of the person on the side-track/loss of the prize money of the contestant with the token on the new path*
  - Peter intentionally *killed the person on the side-track/lost the prize money of the contestant with the token on the new path*
  These were all rated on a rated on a seven point scale (1 *strongly disagree*, to 7 *strongly agree*).
5. How believable is this scenario? Rated on a seven point scale (1 *Not at all believable*, to 7 *Completely believable*).

### Results

Some subjects did not answer all the survey questions. We did not want to create a sample selection bias by only analyzing data from subjects who completed the whole experiment, so the degrees of freedom in the analyses vary depending on how many subjects responded to the question being analyzed.

#### Believability of contexts

Our aim of using the game shows to provide a more realistic context was successful. A Two-Way ANOVA revealed a significant main effect of context on judgments of how believable the scenario was: *F*_(2, 1134)_ = 51.96, *p* < 0.001, η^2^_p_ = 0.084. Tukey *post-hoc* tests revealed that the train context (*M* = 3.16) was significantly less believable than the two game show contexts (large game show *M* = 4.18, small game show *M* = 4.45), both *p* < 0.001. On average, subjects rated all the game show scenarios as believable and the train scenarios as unbelievable. There was also a significant main effect of role, with the onlooker scenarios rated as less believable (*M* = 3.67) than the participant scenarios (*M* = 4.16): *F*_(1, 1134)_ = 19.89, *p* < 0.001, η^2^_p_ = 0.017. There was no significant interaction.

Ratings of “how believable is this scenario?” had a negligible correlation with moral judgment, *r*_(1137)_ = 0.062, *p* = 0.037.

#### Moral judgments

**Figure 1:**
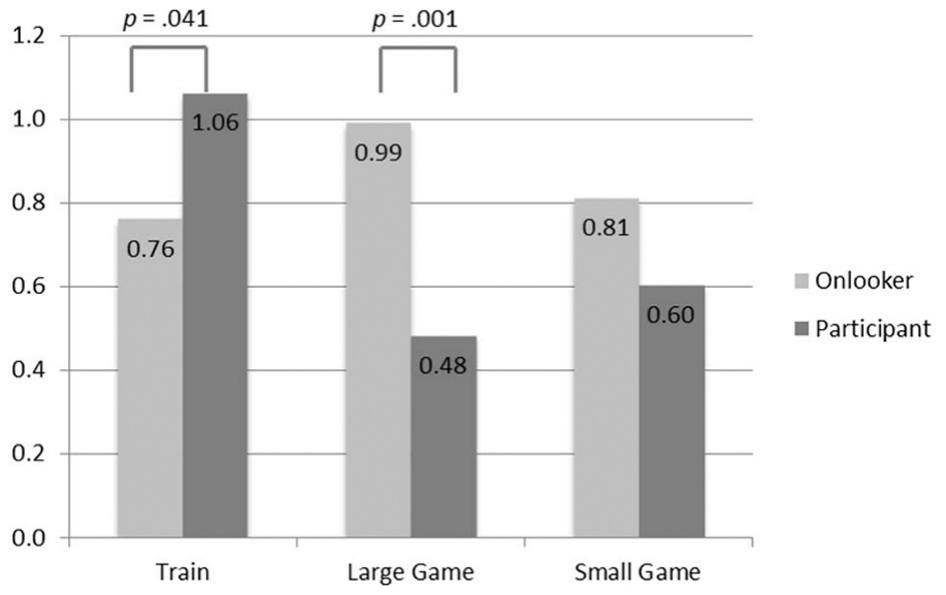
**Mean moral rightness ratings (−3 Definitely wrong to +3 Definitely right)**.

A Two-Way ANOVA showed a significant interaction effect of context and role on rightness judgments: *F*_(2, 1175)_ = 7.98, *p* < 0.001, η^2^_p_ = 0.013 (See Figure 1 for the mean ratings in each scenario). There was no main effect of context, *F*_(2, 1181)_ = 2.33, *p* = 0.098, or of role, *F*_(1, 1181)_ = 2.76, *p* = 0.097.

A simple effects analysis across the contexts shows that there was a difference in the way that subjects rated the action of the participant, *F*_(2, 1175)_ = 9.44, *p* < 0.001, but not of the onlooker, *F*_(2, 1175)_ = 1.34, *p* = 0.261. In the train context, subjects rated the action as more right if the actor was a participant than an onlooker, *F*_(1, 1175)_ = 4.18, *p* = 0.041, but in the large game show context this effect was reversed and the actions of a participant (contestant) were rated as less right than those of an onlooker (audience member)*, F*_(1, 1175)_ = 12.78, *p* < 0.001. There was no effect of role in the small game show context *F*_(1, 1175)_ = 1.93, *p* = 0.165.

**Figure 2:**
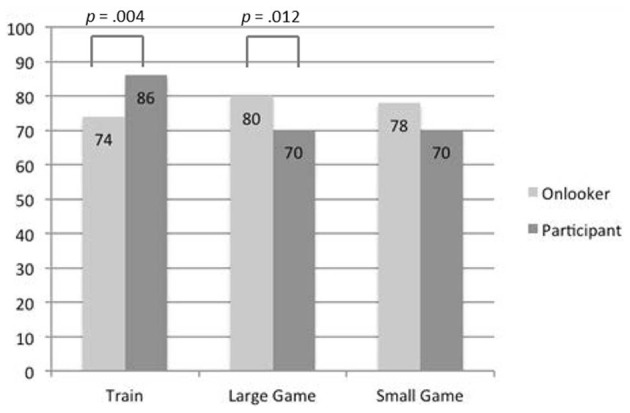
**Percentage of subjects who judged that Peter should pull the lever/press the button**.

Judgments of whether or not Peter *should* pull the lever/press the button differed across the six conditions: χ^2^_(5, 1184)_ = 23.21, *p* < 0.001, ϕ_*c*_ = 0.14. These results are summarized in Figure 2. Pairwise comparisons show that the difference between onlookers and participants is highly significant in the *train* scenario, χ^2^_(1, 400)_ = 8.51, *p* = 0.004, ϕ_*c*_ = 0.146, and the *large game show* scenario, χ^2^_(1, 419)_ = 6.33, *p* = 0.012, ϕ_*c*_ = 0.123, but narrowly failed to reach conventional levels of significance in the *small game show* scenario: χ^2^_(1, 365)_ = 3.38, *p* = 0.066, ϕ_*c*_ = 0.096. In the train scenarios, more people judged that the participant should take the action than the onlooker, but in the game show scenarios more people thought that the onlooker (audience member) should take the action than the participant (contestant). This is the same pattern as the moral judgments.

#### Relation of responsibility, causation, intention, intentionality, and blame to moral judgment

If we look at how each of the factors varies with role and context, using Two-Way ANOVAs, then we find that subjects gave higher ratings for *caused*, *intentionally*, *intended*, and *blame* in the game show scenarios than in the train, all *p* < 0.001 (see Table 1). [The same pattern of results is obtained from a regression analysis. We present partial correlation coefficients in order to make it clear that we make no claims about the direction of causality, which is contested. For opposing views about the direction of causality see Hauser et al. (2007) and Knobe (2010).] There are no significant differences for *responsible*, and no effect of role on any of these factors, or any interaction effects.

**Table 1:** **Mean (and standard deviation) of factor ratings in the scenarios**.

**Context**	**Factor**				
	**Intended**	**Intentionally**	**Caused**	**Blame**	**Responsible**
Train	3.10	2.69	4.31	3.25	0.06
	(2.17)	(2.07)	(2.09)	(2.16)	(1.93)
Large game show	3.68	3.79	4.97	4.10	0.07
	(1.97)	(1.95)	(1.86)	(1.99)	(1.95)
Small game show	3.74	3.85	5.20	4.37	−0.14
	(1.95)	(1.98)	(1.80)	(1.98)	(1.97)

Notes: Responsible rated on a seven point scale (−3 Not at all to +3 Totally), others all rated on a rated on a seven point scale (1 strongly disagree, to 7 strongly agree). For all factors apart from responsibility, means for the game show contexts are different from the mean for the train, p < 0.001. The means for each factor do not differ between the large and small game show contexts.

When we look at the partial correlation coefficients, controlling for the presence of the other variables, we find that only *blame* and *responsible* are correlated with the moral judgment of rightness (see Table 2), but *intended, intentionally* and *caused* are all correlated with *blame* (see Table 3).

**Table 2:** **Partial correlations of the five factors with moral rightness rating**.

**Intended**	**Intentionally**	**Caused**	**Blame**	**Responsible**
−0.038 *p* = 0.206	0.009 *p* = 0.769	0.056 *p* = 0.062	−0.164 *p* < 0.001	0.373 *p* < 0.001

**Table 3:** **Partial correlations of the five factors with each other**.

**Factors**	**Intended**	**Intentionally**	**Cause**	**Blame**	**Responsible**
Intentionally	0.469, *p* < 0.001	1.00			
Caused	−0.064, *p* = 0.031	0.272, *p* < 0.001	1.00		
Blame	0.144, *p* < 0.001	0.121, *p* < 0.001	0.467, *p* < 0.001	1.00	
Responsible	0.038, *p* = 0.197	−0.010, *p* = 0.727	−0.053, *p* = 0.073	0.055, *p* = 0.066	1.00

### Discussion

We found a difference in moral judgment associated with the role of the actor in the scenario, who was the target of the judgment, but the direction of this difference changed depending on the context. In the standard train context, subjects judged that it was more morally right for a passenger, who was already involved in the situation, to turn the train than a bystander, who was an onlooker just passing by. In the game show contexts, it was judged more right for audience members, who were onlookers, than players, who were participating in the quiz, to press the button. Subjects' judgments of what the person in the scenario ought to do followed the same pattern as their moral judgments.

Subjects ascribed a higher degree of causation, intentionality, intention, and blame for the harm in the game show than in the train context. When we tested for relationships between each of these factors and moral judgment, whilst controlling for the other factors, we found that blame was the only factor that both correlated with moral judgment and differentiated the game show from the train context. In turn, the increased blame was related to the actors in the game show being rated higher than those in the train scenario on whether they caused the harm, intended the harm, and brought about the harm to the one intentionally. Hence our data suggest that the relation between moral intuitions and intentionality found by Sinnott-Armstrong et al. (2008) and between moral intuitions and intention, proposed by Mikhail (2007) and found by Hauser et al. (2007), is mediated by differential placing of blame.

Responsibility for taking the action correlated with moral judgments when the other factors were controlled. However, responsibility ratings did not differ between the train and the game show contexts. Thus, Thomson's (1985) suggestion that moral intuitions are related to placing of responsibility is supported, although it does not seem that participants have a greater responsibility to take the action than onlookers.

#### Causes of the reversal

There are two salient differences between the train and the game show scenarios: we changed the context from a train to a game show, and the harmful consequence from death to an economic loss. We think that the reversal of the role effect relates to the change in context, rather than the use of economic harms.

Other studies have replicated trolley results using economic harms. Standard patterns of judgments are seen when economic harms are substituted for mortal harms in hypothetical Side-track and Footbridge scenarios (Gold et al., 2013), and when those judgments are being made about decisions in real Side-track and Footbridge scenarios, involving small economic harms (Gold et al., submitted). Therefore the reversal of the role effect in our hypothetical scenarios seems likely to be related to the change in context, rather than the substituting of economic harm for mortal harms.

Nor do we think that the reversal we found is related to the fact that the game show winnings have been acquired during a show that has not yet ended. One obvious thought is that game show winnings are “funny money,” regarded as not really in the possession of the winner, at least for the duration of the show. However Post et al. (2008) analyzed the behavior of contestants on the television show “Deal or No Deal?” and found that it was consistent with a prospect theory model where decision-makers incorporate expected winnings into their reference point (although the adjustment of the reference point was lagged). This result was not limited to the high stake television game show. It was replicated in classroom experiments with stakes that were 1000 and 10,000 times lower than those on TV. What contestants regard as their current wealth is based on their expectations of how much they will take home, and diminished expectations of winnings represent losses.

The change in context may have affected the causal model that subjects used when representing the problems to themselves—it certainly affected their ascriptions of causation and intentionality—and changing the causal model may affect moral judgments (Spranca et al., 1991; Pizarro et al., 2003; Waldmann and Dieterich, 2007). Whether causation really varies between the train and the game show contexts is a matter for debate. The scenarios were designed so that the explicit causal structures are the same in both contexts. However, the two contexts may have evoked different background assumptions, for example that, in a game show, there are humans involved in running the show who have a causal role in the outcomes and who may bear some blame, whereas in the train context there is no obvious person who is causally responsible or to blame for the malfunction of the train.

The two contexts may also have differed with respect to which agents are perceived to have the right to cause the harm: the participant (passenger) in the train context but the onlooker (audience member) in the game show. People who say that they would not turn the trolley give reasons including not having the right to decide and not wanting to be responsible for someone's death (Gold et al., 2014). Similarly, people who say that they would not vaccinate their child if there was a risk of death cite being responsible for any negative consequence of the action (Ritov and Baron, 1990). (Being responsible for the bad consequence is subtly different from the question we asked, about being responsible for acting, and having the right to act is clearly different from having the responsibility—or duty—to act). Having rights and responsibilities can be connected to the social roles we occupy (Baron, 1996), so the right thing to do in dilemmas with similar structures can be sensitive to context.

## Study 2

In our second study we operationalized the small game show in a laboratory setting. We conducted a quiz, which subjects either took part in (players) or watched (audience), with monetary prizes for all players who correctly completed more than 15 out of 20 questions. Once at least six players had answered enough questions correctly to collect prizes, we paused the quiz and threatened to knock out five of them, who would lose their winnings. The actor had to decide whether to press a button to keep them in, with the side-effect that we would knock one, different player out of the quiz, who would lose his or her winnings. We varied whether the actor was a player or an audience member.

This enabled us to investigate whether the role of the actor, who was the target of judgment, would affect judgments in a real life scenario (Target Role: *target player* vs. *target audience*). We also varied the role of the subjects who were making the judgments, (Subject Role: *player* vs. *audience*).

Since we had to have some subjects making the decisions that were being judged, we were also able to observe behavior and to compare the judgments of *actors*, who made decisions and judged the morality of their own decision, with those of *observers*, who made judgments about the action of an actor (Decision Making Power: *actor* vs. *observer*). Actors always made judgments about their own action, so we did not cross subject role and target role for actors.

In Study 1, our questions were all about a third person (“should Peter/a passenger press the button?”). In Study 2, the actors were asked about their own actions and the observers were asked about a third person (“should the player/audience member press the button?”). Hence the nearest equivalent to the difference investigated in Study 1 is when the subject is an observer and the target role is varied, target player vs. target audience.

### Methods

#### Subjects

There were 202 subjects, 105 men, and 97 women. They were aged between 18 and 56 years (*M* = 22.02, *SD* = 6.11). Subjects were recruited through the University of Leicester's online e-bulletin, which goes out to staff and students. They were tested in groups of 35–40.

#### Procedure and materials

**Figure 3:**
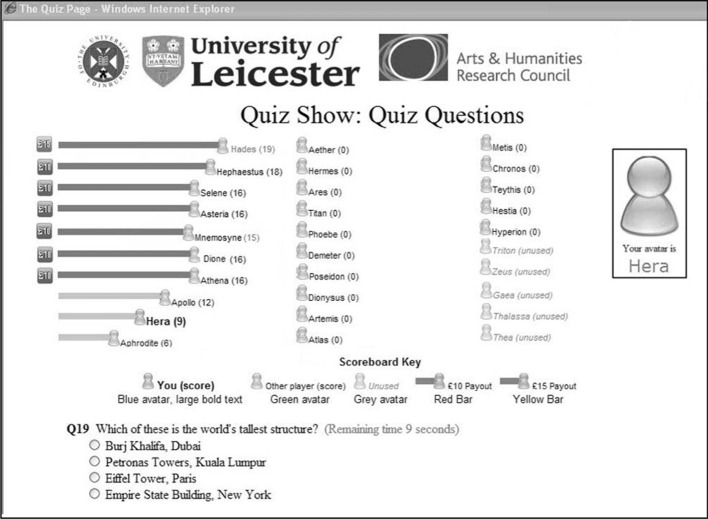
**Example of a subject's monitor displaying the questions and the progress of the quiz show subjects**.

Subjects sat at computer terminals in one large room and took part in a quiz show (see Figure 3). We randomly selected 60% of the subjects to be players, taking part in a general knowledge quiz, and they were assigned pseudonyms. The other 40% were the audience, watching the quiz on their screens. The audience saw the questions in real time and watched the progress of avatars, representing the players, moving across the screen. Players who answered fifteen questions correctly entered the winning zone. Subjects were told that any player who was in the winning zone at the end of the quiz would get £10, and any player who correctly answered nineteen or twenty questions would receive £15. At the end of the experiment, players were paid their winnings, or a £5 show-up fee if they won nothing. Audience members were paid £5 for their participation.

Once six players had entered the winning zone, the quiz stopped. The six players in the zone received a screen message saying “please wait.” These players took no decisions and thus these 36 subjects provided no further data to the experiment. Other players received a screen message, whose content depended on the condition that they were in.

Actors (both players and audience members) received the following message:

Five of the players who are in the winning zone are about to be knocked out of the game by the experimenter and will each lose their £10 cash winnings. You can stop the five from losing their winnings by pressing the button below. However, in that case the experimenter will knock out a different player who is in the winning zone, and the one player will lose his/her £10 cash.

Those actors who were players were also told:

Whether or not you press the button won't affect your winnings. If you are in the winning zone, then you are not one of the players who is affected by this decision.

Observers received the following message, phrases in italics varied, depending on whether the actor whose behavior was being judged was a player or an audience member:

Five of the players who are in the winning zone are about to be knocked out of the game by the experimenter and will each lose their £10 cash winnings. *Another of the players/An audience member* is being given the option of pressing a button to stop the five from losing their winnings. However, in that case the experimenter will knock out a different player who is in the winning zone, and the one player will lose his/her £10 cash.

In addition, those who were judging a player were also told:

Whether or not the player presses the button won't affect his/her winnings. If s/he is in the winning zone, then s/he is not one of the players who is affected by the decision and s/he knows this.

Actors then had 60 s to decide whether or not to push the button. Observers were asked how strongly they agreed with the statement: The *player/audience member* should press the button, rated on a nine point scale (1 *Strongly disagree* to 9 *Strongly agree*).

Subjects were then asked to indicate how wrong or how right it would have been to press the button, on a scale from 1 (*Definitely wrong*) to 9 (*Definitely right*).

At the beginning of the experiment, subjects had been told that “In this experiment some decisions will affect other subjects' payments and some will not,” and one randomly selected actor's decision was implemented to see who got knocked out, the one player or the five.

### Results

Among actors, the decision to press the button or not was unaffected by whether the person given the choice was a player (78.57% pressed it) or audience member (76.67% pressed it), χ^2^_(1, 58)_ = 0.030, *p* = 0.862, ϕ_*c*_ = 0.023. Thus it seems that being a part of the quiz did not increase the proportion of people willing to press the button compared to the people who were merely watching it.

**Figure 4:**
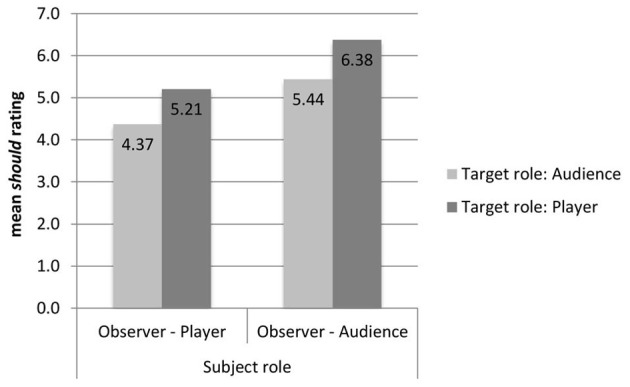
**Mean rating of “The player/audience member should press the button” (1 Strongly disagree to 9 Strongly agree)**.

Observers' judgments of whether the actor should press the button were affected by their own roles, as player or audience member (see Figure 4). We examined the mean ratings of whether the observers thought that the actor should press the button (1 *strongly disagree* to 9 *strongly agree*) as the dependent variable in a Two-Way ANOVA with Subject Role (*player* vs. *audience*) and Target Role (*target player* vs. *target audience*) as independent variables. There was a main effect of Subject Role, with audience members agreeing more strongly that the actor should press the button (*M* = 5.92) than the players (*M* = 4.79), *F*_(1, 104)_ = 5.12, *p* = 0.026, η^2^_*p*_ = 0.047. On average, audience members believed that the actor should press the button (mean rating above 5), but players did not (mean rating below 5). Regarding the Target Role, there was a trend for the player to be judged higher than the audience member (5.80 vs. 4.91), *p* = 0.075, η^2^_*p*_ = 0.030. Our subjects believed, on average, that the player should press the button (mean rating above 5), but that the audience member should not (mean rating below 5). Note that this trend is in the opposite direction of the effect we found in Study 1. There was no interaction between the two factors.

**Figure 5:**
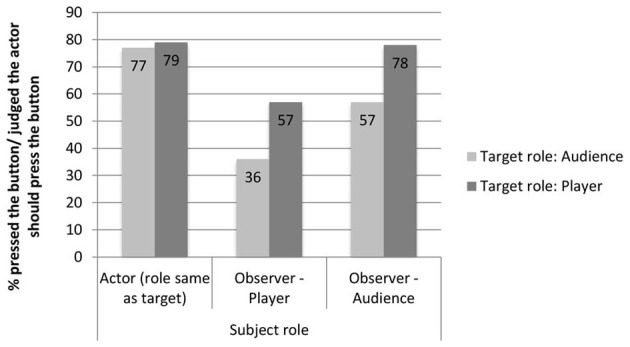
**Percentage of actors who pressed the button, and percentage of observers who judged that their target should press the button, broken down by the role of the subject and the role of the target**.

If we group the observers into those who judged that the actor should not take the action (those who gave a rating from 1 to 4) and those who judged that s/he should (rating from 6 to 9), we can more easily compare the data to both the actions of the actors (see Figure 5).

Actors were more likely to take action than observers were to judge that they should. The only condition where judgments about what should be done corresponded to what was actually done was that of the audience members whose judgment targeted a player.

There was also an effect due to the role of the person making the judgment. Observers' judgments of whether or not the target should press the button differed across the four conditions: χ^2^_(3, 91)_ = 8.07, *p* = 0.044, ϕ_*c*_ = 0.298. The observer's judgments differ depending on the role of the subject, (67.4% of the observers who are audience members say the target should press the button and 46.7% of the observer players say the target should press the button), χ^2^_(1, 91)_ = 3.99, *p* = 0.046 ϕ_*c*_ = 0.209, and depending on the target of judgment, (46.7% of the observers think that the audience members should press the button and 67.4% think that players should press the button), χ^2^_(1, 91)_ = 3.99, *p* = 0.046, ϕ_*c*_ = 0.209. This grouping corroborates the pattern found in the ratings: there was a clear reversal of the target-role effect found in the hypothetical game shows in Study 1. In Study 1, subjects were more likely to say that the audience members should press the button, whereas in Study 2, the observers (whose positions correspond most closely to the subjects in Study 1) are more likely to say that the player should take action than the audience member.

The rating of how right or wrong pressing the button was did not vary according to whether the subject was an actor or an observer (*M* = 4.22 vs. 4.45), *F*_(1, 160)_ = 0.13, *p* = 0.722, or with Subject Role, audience or player, (*M* = 4.10 vs. 4.65), *F*_(1, 160)_ = 2.71, *p* = 0.101, or Target Role, audience or player, (*M* = 4.48 vs. 4.27), *F*_(1, 160)_ = 1.19, *p* = 0.276, nor was there any interaction between these factors. In every condition, the average right-wrong judgment fell on the “wrong” side of the scale yet, in three out of four of the observer conditions, a majority of subjects judged that the actor should press the button, and a large majority of actors pressed the button.

### Discussion

In Study 2, we operationalized the small game show from Study 1, and the target-role effect in “should” judgments reversed back to being in the same direction as the judgments in the hypothetical train context: when the target was a player more subjects thought that s/he should press the button than when the target was an audience member. Our results are consistent with other evidence that real moral decisions can dramatically contradict moral choices made in hypothetical scenarios (FeldmanHall et al., 2012b).

A key difference between our real and realistic scenarios is that actually being in the scenario may have evoked a “hot” affective state whereas contemplating the same hypothetical scenario is done in an affectively “cold” state. Differences in affective states between real and hypothetical scenarios could cause judgments and behavior to be different (Kühberger et al., 2002; Kang and Camerer, 2013). People are probably not even aware that their judgments would differ in real and hypothetical scenarios because there is a “hot/ cold empathy gap,” where people mispredict the effect of their affective state on their preferences and behavior (Loewenstein, 2005). Yet, in a real task, manipulating whether participants are in “hot” or “cold” states affects behavior, with the “hot” version being associated with more risk taking and poorer information use (Figner et al., 2009).

The importance of affective states is supported by neuroscientific evidence. Real and hypothetical moral decisions differentially recruit neural circuitry, with hypothetical moral decisions eliciting activity in neural circuits that are involved in imagination, whilst real moral decisions activate the amygdala, which is crucial for social and affective processes (FeldmanHall et al., 2012a). There is also increased activity in the amygdala when subjects are presented with stories that narrate their own intentional violation of social norms, compared to violations by others; this has been linked to enhanced emotional responses (Berthoz et al., 2006).

Others have stressed the importance of emotional reactions in trolley problems (e.g., Greene et al., 2001) and “hot” affect may connect our outlandish and real scenarios. The outlandish trolley scenario may elicit a strong emotional response because the hypothetical outcomes involve deaths; the real scenario may evoke an emotional response because the small harms will actually occur. So both the outlandish and the real scenarios may have provoked a more emotional response than the realistic scenarios. Thus we observed similar patterns of responses in the outlandish and the real scenarios and a different pattern in the realistic scenarios.

There was also a difference in judgments depending on the role of the subject making the judgment: audience members were more likely to judge that the actor should press the button. Audience members and players might have differentially empathized with the one player who risked being knocked out, with players being more likely to think “what if it were me?” Interestingly, when observers judge people in the same role as themselves—when players judge players and audience members judge audience members—57% of both groups think that the actor should press the button. It is when these two groups judge people from a different role that stark differences appear. When audience members judge players 78% of them think the player should press the button, a figure that matches almost precisely the number of actors who actually do take action. In contrast, only 36% of the players think that an audience member should press the button to save the five from losing their money, thus indicating that the majority of players feel that the audience members should stay out of the situation and not intervene.

Actors consistently pressed the button, and more actors pressed the button than observers said should press the button. We did not ask actors for their judgment about what they should do, as it risked merely eliciting self-justificatory answers. If the observers' judgments are indicative of what actors thought they should do, then many actors pressed the button despite thinking that they ought not to. This is a case of weakness of will. Alternatively, if actors acted in line with their judgments about what they ought to do, then having the power to make a decision affects one's judgment about what ought to be done. In either case, it appears that asking an observer what should be done gets different results from observing actual actions.

Despite the difference in opinions about what should be done amongst observers, there are no differences in moral judgments between the groups in Study 2. Different patterns of hypothetical choice and moral judgments have also been found by Tassy et al. (2013), who hypothesize that this occurs because choice and judgment are the results of different psychological processes; and different patterns of actual choice and moral judgments have been found by Gold et al. (submitted), who suggest that their subjects found that the normatively relevant factors for whether or not to press the button were not exhausted by its moral right and wrongness. There may be pragmatic factors in play.

## General discussion

We found differences in moral judgments between outlandish and realistic hypothetical scenarios, and between judgments made in hypothetical scenarios vs. the same scenarios operationalized in real-life. Of course, showing that there are differing responses cannot tell us which responses are “correct” or which type of scenarios we should study (Elqayam and Evans, 2011). But we can outline some of the advantages and disadvantages of each approach.

Researchers may choose to use outlandish artificial dilemmas, rather than realistic ones, in order to isolate the dimensions that are of theoretical interest (Hauser et al., 2007). Real life scenarios are usually complex, so isolating dimensions of interest generally necessitates using outlandish scenarios. Some researchers see subjects' lack of familiarity with the outlandish scenarios as a further point in their favor, because it removes some of the social and personal factors that might otherwise influence responses (Hauser et al., 2007). But both of these supposed benefits are contested, particularly when dilemmas are used in ethics. There is a move, especially in medical ethics, to see moral dilemmas as occurring within a broader narrative, so their resolution requires moral imagination and a more holistic engagement with all the features of the case (Hunter, 1996; London, 2001). There are also arguments that we can be most sure of our moral judgments when we contemplate complicated and familiar cases: either particular paradigm cases, such as landmark legal cases (Jonsen and Toulmin, 1988), or familiar situations (Woodward and Allman, 2007).

Real and hypothetical dilemmas may put subjects in different affective states (Kühberger et al., 2002; Kang and Camerer, 2013). There is disagreement whether subjects should be in “hot” or “cold” states when moral judgments are elicited. Real-life moral cognition is hot cognition and, if hot and cold judgments differ, especially if they involve different brain systems, it follows that psychological studies of moral cognition would benefit from being done in ecologically valid settings (Casebeer, 2003; Moll et al., 2005). However, when judgments are used for philosophical purposes, it has been argued that we should be wary of judgments that are driven by “ ‘alarm bell’ emotion” (Greene, 2007, p.63), which suggests privileging “cold” judgments.

Researchers should bear in mind that whether scenarios are outlandish, realistic, or real may affect moral judgments. But which type of scenarios is most appropriate to use may depend on the nature and purpose of the study. Furthermore, a complete understanding of the significant differences reported in our experiments will, of course, require a great deal more research, and the potential explanations are myriad. It is even possible, following a suggestion made by Skinner (1985) in a generalized critique of cognitive science, that the differences could be explained by people's application of patterns of behavior learnt under contingencies of reinforcement in analogous situations experienced in everyday life. However, such purely behavioral explanations are bound to exist alongside interpretations in cognitive and ethical terms.

### Conflict of interest statement

The authors declare that the research was conducted in the absence of any commercial or financial relationships that could be construed as a potential conflict of interest.

## Footnotes

^1^We had originally hoped to compare Side-track and Footbridge, but we struggled to come up with any real life examples of dilemmas with a similar structure to Footbridge—grist to the mill of Woodward and Allman's (2007) argument.

## References

[B1] Andreoni J. Brown P. M. Vesterlund L. (2002). What makes an allocation fair? Some experimental evidence. Games Econ. Behav. 40, 1–24 10.1006/game.2001.0904

[B2] Andreoni J. Miller J. (2002). Giving according to GARP: an experimental test of the consistency of preferences for altruism. Econometrica 70, 737–753 10.1111/1468-0262.00302

[B3] Baron J. (1996). Do no harm, in Codes of Conduct: Behavioral Research into Business Ethics, eds Messick D. M. Tenbrunsel A. E. (New York, NY: Russell Sage Foundation), 197–213

[B4] Berg J. Dickhaut J. McCabe K. (1995). Trust, reciprocity, and social history. Games Econ. Behav. 10, 122–142 10.1006/game.1995.1027

[B5] Berthoz S. Grezes J. Armony J. Passingham R. Dolan R. (2006). Affective response to one's own moral violations. Neuroimage 31, 945–950 10.1016/j.neuroimage.2005.12.039 16490367

[B6] Bolton G. Ockenfels A. (2000). ERC: a theory of equity, reciprocity, and competition. Am. Econ. Rev. 90, 166–193 10.1257/aer.90.1.166

[B51a] Casebeer W. D. 2003 Moral cognition and its neural constituents. Nat. Rev. Neurosci. 4, 840–847 10.1038/nrn1223 14523383

[B8] CNN U.S. (2003). Runaway freight train derails near Los Angeles. CNN U.S. Available online at: http://articles.cnn.com/2003-06-20/us/train.derails_1_derailment-freight-cars-runaway-freight-train?_s=PM:US

[B9] Cushman F. A. Young L. Hauser M. D. (2006). The role of conscious reasoning and intuition in moral judgments: testing three principles of harm. Psychol. Sci. 17, 1082–1089 10.1111/j.1467-9280.2006.01834.x 17201791

[B10] Elqayam S. Evans J. S. B. (2011). Subtracting “ought” from “is”: descriptivism versus normativism in the study of human thinking. Behav. Brain Sci. 34, 233–248 10.1017/S0140525X1100001X 22000212

[B11] Fehr E. Schmidt K. M. (1999). A theory of fairness, competition, and cooperation. Q. J. Econ. 114, 817–868 10.1162/003355399556151

[B12] Fehr E. Schmidt K. M. (2006). The economics of fairness, reciprocity and altruism – experimental evidence and new theories, in Handbook of the Economics of Giving, Altruism and Reciprocity, Vol. 1, eds Kolm S.-C. Ythier J. M. (North Holland: Elsevier), 615–691 10.1016/S1574-0714(06)01008-6

[B13] FeldmanHall O. Dalgleish T. Thompson R. Evans D. Schweizer S. Mobbs D. (2012a). Differential neural circuitry and self-interest in real vs. hypothetical moral decisions. Soc. Cogn. Affect. Neurosci. 7, 743–751 10.1093/scan/nss069 22711879 PMC3475363

[B14] FeldmanHall O. Mobbs D. Evans D. Hiscox L. Navrady L. Dalgleish T. (2012b). What we say and what we do: the relationship between real and hypothetical moral choices. Cognition 123, 434–441 10.1016/j.cognition.2012.02.001 22405924 PMC3355304

[B15] Figner B. Mackinlay R. J. Wilkening F. Weber E. U. (2009). Affective and deliberative processes in risky choice: age differences in risk taking in the Columbia Card Task. J. Exp. Psychol. Learn. Mem. Cogn. 35, 709–730 10.1037/a0014983 19379045

[B16] Foot P. (1967). The problem of abortion and the doctrine of double effect. Oxford Rev. 5, 5–15

[B17] Gigerenzer G. Todd P. the ABC Research Group (1999). Simple Heuristics that Make us Smart. New York, NY: Oxford University Press

[B19] Gold N. Colman A. M. Pulford B. D. (2014 Cultural differences in response to real-life and hypothetical trolley problems. Judgm. Decis. Mak. 9, 65–76 Available online at: journal.sjdm.org/12/121101/jdm121101.pdf

[B20] Gold N. Pulford B. D. Colman A. M. (2013). Your money or your life: comparing judgments in trolley problems involving economic and emotional harms, injury and death. Econ. Philos. 29, 213–233 10.1017/S026626711300020514650065

[B22] Greene J. D. Sommerville R. B. Nystrom L. E. Darley J. M. Cohen J. D. (2001). An fMRI investigation of emotional engagement in moral judgment. Science 293, 2105–2108 10.1126/science.1062872 11557895

[B23a] Greene J. D. (2007). The secret joke of Kant's soul, in Moral Psychology, The Neuroscience of Morality: Emotion, Disease, and Development, Vol. 3, ed Sinnott-Armstrong (Cambridge, MA: MIT Press).

[B23] Hauser M. D. Cushman F. A. Young L. Kang-Xing Jin R. Mikhail J. (2007). A dissociation between moral judgments and justifications. Mind Lang. 22, 1–21 10.1111/j.1468-0017.2006.00297.x

[B24] Hunter K. M. (1996). Narrative, literature, and the clinical exercise of practical reason. J. Med. Philos. 21, 303–320 10.1093/jmp/21.3.303 8803811

[B25] Jonsen A. R. Toulmin S. (1988). The Abuse of Casuistry: A History of Moral Reasoning. Berkeley, CA: University of California Press

[B26] Kamm F. M. (1989). Harming some to save others. Philos. Stud. 57, 227–260 10.1007/BF00372696

[B27] Kamm F. M. (1996). Morality, Mortality, Volume II: Death and Whom to Save from it. New York, NY: Oxford University Press

[B28] Kang M. J. Camerer C. F. (2013). fMRI evidence of a hot-cold empathy gap in hypothetical and real aversive choices. Front. Neurosci. 7:104 10.3389/fnins.2013.00104 23772205 PMC3677130

[B29] Knobe J. (2010). Action trees and moral judgment. Top. Cogn. Sci. 3, 555–578 10.1111/j.1756-8765.2010.01093.x 25163876

[B30] Knutson K. M. Krueger F. Koenigs M. Hawley A. Escobedo J. R. Vasudeva V. (2010). Behavioral norms for condensed moral vignettes. Soc. Cogn. Affect. Neurosci. 5, 378–384 10.1093/scan/nsq005 20154053 PMC2999756

[B31] Kühberger A. Schulte-Mecklenbeck M. Perner J. (2002). Framing decisions: hypothetical and real. Organ. Behav. Hum. Decis. Process. 89, 1162–1175 10.1016/S0749-5978(02)00021-3

[B32] Loewenstein G. (2005). Hot-cold empathy gaps and medical decision making. Health Psychol. 24, S49–S56 10.1037/0278-6133.24.4.S49 16045419

[B33] London A. J. (2001). The independence of practical ethics. Theor. Med. Bioeth. 22, 87–105 10.1023/A:1011403909450 11437274

[B34] Mikhail J. (2007). Universal moral grammar: theory, evidence, and the future. Trends Cogn. Sci. 11, 143–152 10.1016/j.tics.2006.12.007 17329147

[B35] Moll J. Zahn R. de Oliveira-Souza R. Krueger F. Grafman J. (2005). Opinion: the neural basis of human moral cognition. Nat. Rev. Neurosci. 6, 799–809 10.1038/nrn1768 16276356

[B36] Nadelhoffer T. Feltz A. (2008). The actor-observer bias and moral intuitions: adding fuel to Sinnott-Armstrong's fire. Neuroethics 1, 133–144 10.1007/s12152-008-9015-7

[B37] Pizarro D. A. Uhlmann E. Bloom P. (2003). Causal deviance and the attribution of moral responsibility. J. Exp. Soc. Psychol. 39, 653–660 10.1016/S0022-1031(03)00041-6

[B38] Post T. Van den Assem M. J. Baltussen G. Thaler R. H. (2008). Deal or no deal? Decision making under risk in a large-payoff game show. Am. Econ. Rev. 98, 38–71 10.1257/aer.98.1.3819920882

[B39] Pulford B. D. Colman A. M. Gold N. (2012). Investigating the effects of framing in trolley problems, in Keynote Paper Presented at the Experiments in Ethical Dilemmas Workshop (London).

[B40] Quinn W. S. (1989). Actions, intentions, and consequences: the doctrine of doing and allowing. Philos. Rev. 98, 287–312 10.2307/2185021 11659159

[B41] Ritov I. Baron J. (1990). Reluctance to vaccinate: omission bias and ambiguity. J. Behav. Decis. Mak. 3, 263–277 10.1002/bdm.3960030404

[B42] Rozyman E. Baron J. (2002). The preference for indirect harm. Soc. Justice Res. 15, 165–184 10.1023/A:1019923923537

[B43] Schaich Borg J. Hynes C. Van Horn J. Grafton S. Sinnott-Armstrong W. (2006). Consequences, action, and intention as factors in moral judgments: an fMRI investigation. J. Cogn. Neurosci. 18, 803–817 10.1162/jocn.2006.18.5.803 16768379

[B44] Sinnott-Armstrong W. Mallon R. McCoy T. Hull J. G. (2008). Intention, temporal order, and moral judgments. Mind Lang. 23, 90–106 10.1111/j.1468-0017.2007.00330.x

[B45] Skinner B. F. (1985). Cognitive science and behaviorism. Br. J. Psychol. 76, 291–301 10.1111/j.2044-8295.1985.tb01953.x 4041702

[B46] Spranca M. Minsk E. Baron J. (1991). Omission and commission in judgment and choice. J. Exp. Soc. Psychol. 27, 76–105 10.1016/0022-1031(91)90011-T 23071678

[B47] Tassy S. Oullier O. Mancini J. Wicker B. (2013). Discrepancies between judgment and choice of action in moral dilemmas. Front. Psychol. 4:250 10.3389/fpsyg.2013.00250 23720645 PMC3655270

[B48] Thomson J. J. (1976). Killing, letting die, and the trolley problem. Monist 59, 204–217 10.5840/monist197659224 11662247

[B49] Thomson J. J. (1985). The trolley problem. Yale Law J. 94, 1395–1415 10.2307/796133

[B50] Waldmann M. R. Dieterich J. H. (2007). Throwing a bomb on a person versus throwing a person on a bomb: intervention myopia in moral intuitions. Psychol. Sci. 18, 247–253 10.1111/j.1467-9280.2007.01884.x 17444922

[B51] Woodward J. Allman J. (2007). Moral intuition: its neural substrates and normative significance. J. Physiol. Paris 101, 179–202 10.1016/j.jphysparis.2007.12.003 18280713

